# Intragenomic riboregulatory interaction modulates the conformation of the hepatitis C virus 3′X RNA

**DOI:** 10.1016/j.jbc.2026.113264

**Published:** 2026-06-19

**Authors:** Ethan P. Rogers, Parker D. Sperstad, Erik D. Holmstrom

**Affiliations:** 1 Department of Molecular Biosciences, University of Kansas, Lawrence, Kansas, USA; 2 Department of Chemistry, University of Kansas, Lawrence, Kansas, USA

**Keywords:** hepatitis C virus, untranslated region, 5BSL3.2, 3′X, FRET, fluorescence, single-molecule, RNA structure, RNA binding, RNA folding, riboregulatory

## Abstract

The 3′X RNA of the hepatitis C virus (HCV) is a highly conserved noncoding element required for viral replication. This RNA is known to make intragenomic interactions with another highly conserved element, 5BSL3.2, located in the open reading frame of the HCV genome. This RNA–RNA interaction is part of a riboregulatory network that is hypothesized to control various aspects of viral replication. Here, we used single-molecule FRET to characterize the binding mechanism of this riboregulatory interaction. Binding affinities and equilibrium constants were determined under a wide range of experimental conditions. Our results suggest that 5BSL3.2 differentially associates with the two conformations populated by the first 55 nucleotides of 3′X (3′X55), modestly favoring one bound conformation over the other. These findings shed new light on the structural nature of this interaction and appear to support an emerging hypothesis wherein the presence or absence of this intragenomic interaction influences viral translation and replication.

Hepatitis C is a liver inflammatory disease that infects ∼ 1 million people every year (1), often resulting in chronic liver cirrhosis and cancer. This disease is caused by the hepatitis C virus (HCV), a lipid-enveloped particle ∼ 50 nm in diameter (2, 3) that contains a positive-sense, single-stranded RNA genome of ∼ 9600 nucleotides. The HCV genome is highly structured and can be partitioned into three distinct regions: the 5′ untranslated region (5′UTR), the polyprotein open-reading frame (ORF), and the 3′ untranslated region (3′UTR). The 5′UTR contains an internal ribosome entry site (IRES), which facilitates cap-independent translation of the genome by directly recruiting the 40S ribosomal subunit (4, 5). At over 9000 nucleotides long, the ORF is the largest region of the HCV genome and encodes a 10-protein polyprotein. Lastly, the 3′UTR terminates the genome with a domain referred to as 3′X; a highly conserved, 98-nucleotide non-coding element required for viral replication (6, 7).

Unfortunately, the molecular structure and biochemical function of 3′X have remained elusive for many decades. Previous experimental and computational analyses (8, 9, 10, 11, 12) have revealed that the first 55 nucleotides of this sequence primarily adopt two distinct conformations: 3′X55a and 3′X55b (Fig. 1*A*). Importantly, 3′X55 is known to engage in interactions with another highly conserved RNA element in the genome, 5BSL3.2 (11, 13, 14, 15) (Fig. 1*B*). This 48-nucleotide cis-regulatory element forms a stem-loop structure near the end of the ORF in the region coding for the RNA-dependent RNA polymerase, NS5B. In addition to its ability to interact with 3′X55, 5BSL3.2 has also been shown to interact with the IRES, which modulates translation of the viral polyprotein (4, 16). The interactions among these three structured elements in the viral genome are hypothesized to form a riboregulatory network that may prevent genomic competition between ribosomes and polymerases during translation and replication (17, 18) (Fig. 1*C*). The functional importance of this riboregulatory network has been demonstrated in several previous genetic studies of subgenomic replicon systems (13, 16, 19, 20, 21), where mutations that disrupt these intragenomic interactions significantly affect reporter expression. Notably, even small, targeted mutations in these interacting RNA elements can have detrimental effects that can be restored by compensatory mutations (13, 19). Together, the results of these studies highlight the importance of the interactions between 3′X55 and 5BSL3.2 in both IRES-mediated translation and NS5B-mediated replication.

**Figure 1:**
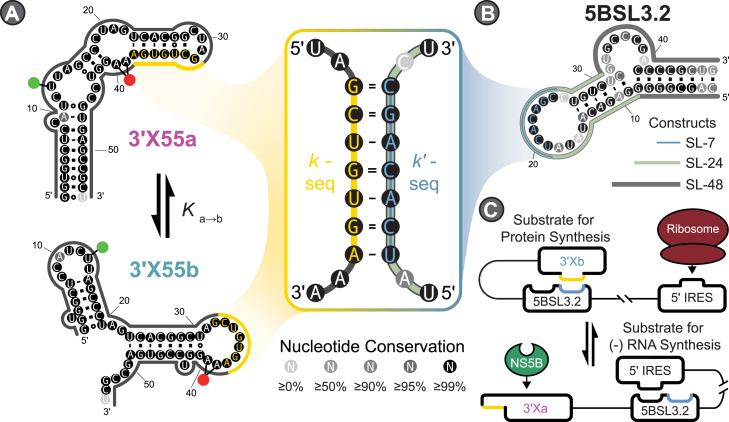
**The intramolecular conformational equilibrium of 3′X55 and its interactions with 5BSL3.2.***A*, 3′X55 adopts two distinct monomeric conformations (3′X55a and 3′X55b) that slowly interconvert (12). Importantly, this very highly conserved RNA contains the 7-nucleotide (nt) *k*-sequence (*yellow*). The secondary structures for 3′X55a and 3′X55b were taken from previously published reports (8, 9). *B*, 5BSL3.2 is a slightly less highly conserved 48-nt sequence found within the coding region of NS5B. Previous work has shown that 5BSL3.2 can engage in intragenomic interactions with both the IRES (16, 21) and 3′X55 (13). The latter occurs *via* a complementary kissing-loop interaction between the 7-nt *k′*-sequence (*blue*) of 5BSL3.2 and the *k*-sequence (*yellow*) of 3′X55. In this work, we utilized three variants of 5BSL3.2 to study the structural context of this *k-k′* interaction: SL-48, SL-24, and SL-7 (Table S1). *C*, diagram of a possible riboregulatory mechanism involving all three regions of the HCV genome, adapted from (12). If the *k-k′* interaction forms, the IRES is then free for ribosome recruitment, allowing for protein synthesis. However, if the IRES binds 5BSL3.2, then the *k-k′* interaction cannot form, and the 3′-end of the RNA is available for (−) RNA synthesis *via* NS5B.

To further assess the merits of such a riboregulatory network, we set out to biochemically characterize structural and energetic aspects of the heterodimeric interaction between 3′X55 and 5BSL3.2. This intragenomic kissing-loop interaction (Fig. 1, *A* and *B*) natively occurs between two complementary 7-nucleotide sequences in the HCV genome: the *k*-sequence of 3′X55 and the *k′*-sequence of 5BSL3.2 (11, 15, 19). Importantly, the accessibility of the *k-*sequence depends on the intramolecular conformational equilibrium of 3′X55 (12). Specifically, 3′X55a sequesters most of the *k-*sequence within a paired stem, while 3′X55b presents the entire *k-*sequence within an apical loop (Fig. 1*A*). Curiously, previous NMR studies yield differing conclusions regarding the structural context of this interaction (Fig. S1): some suggest that the *k-*sequence of 3′X55a locally melts to form a complex with 5BSL3.2 (14, 15), and others propose that this complex forms *via* direct interactions with the exposed *k-*sequence of 3′X55b (11).

Here, we used Förster Resonance Energy Transfer (FRET) spectroscopy to monitor how the intramolecular conformational equilibrium of fluorescently labeled 3′X55 (Fig. 1*A*) is influenced by intermolecular interactions with unlabeled 5BSL3.2 (Fig. 1*B*). FRET is a distance-dependent photophysical process that provides a non-radiative pathway for energy transfer between an excited donor fluorophore and an adjacent acceptor fluorophore. This phenomenon is often exploited to monitor biomolecular distances in a wide variety of solution conditions (22, 23). With concentrations of fluorescently labeled 3′X55 at approximately 100 pM, our single-molecule FRET experiments allowed us to study several conformationally heterogeneous subpopulations of this RNA while preventing homodimerization (12, 24). Using this approach, we have been able to describe several structural and energetic aspects of this riboregulatory interaction between 3′X55 and 5BSL3.2.

## Results

Nucleotide conservation is a common signature of functionally significant sequences within the genomes of all lifeforms. Recently, a multiple-sequence alignment of representative HCV genomes demonstrated that 3′X and 5BSL3.2 are particularly well conserved and likely form an intragenomic kissing-loop interaction (25). Here, we have independently assessed the conservation of these specific regions of the HCV genome using all available *Hepacivirus hominis* sequences in the NCBI nucleotide database and arrived at the same conclusions. Our bioinformatic analysis indicates that the complementary sequences that form this riboregulatory interaction (*i.e.*, the *k*- and *k′*-sequences) are > 99% conserved (Fig. 1) and bolsters the notion that the interaction between 3′X and 5BSL3.2 may be important for viral replication (12, 19, 25, 26).

**Figure 2:**
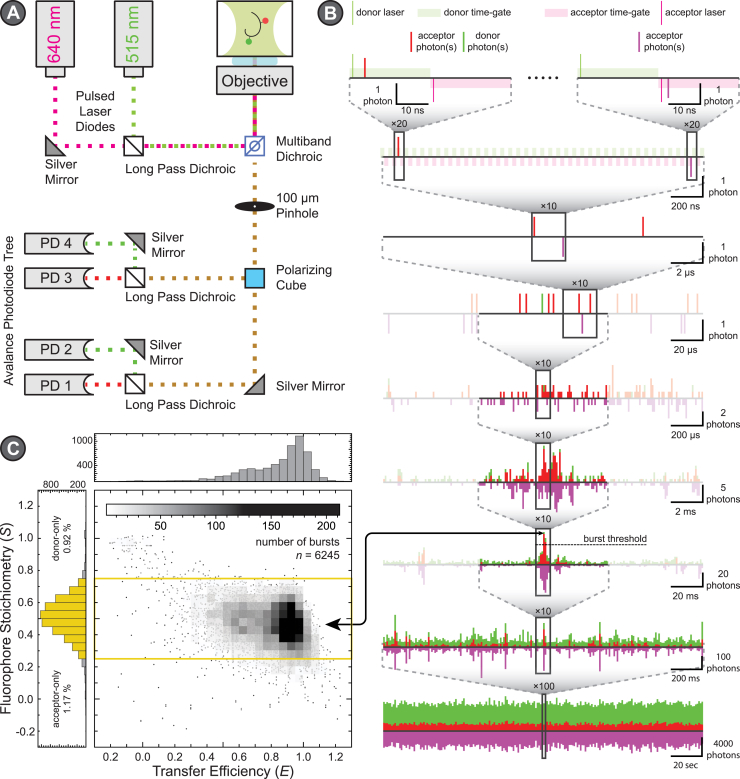
**Single-molecule FRET.***A*, simplified diagram of our confocal fluorescence microscopy system showcasing essential components. *B*, two picosecond laser diodes are operated in a pulsed (20 MHz) and interleaved fashion to alternately excite the donor and acceptor fluorophores. Individual photon arrival times are recorded with 16 ps resolution over the course of 10-min. When the data are binned at 1 ms (third from *bottom*), individual bursts of fluorescence can be easily identified for downstream analysis. *C*, bursts can be further filtered by fluorophore stoichiometry (*S*), which distinguishes FRET-active bursts from donor-only and acceptor-only bursts, and transfer efficiency (*E*), which reports on the inter-fluorophore distance of the fluorescently labeled RNAs.

In this report, we study how the structure of the first 55-nucleotides of 3′X (3′X55) changes upon binding to 5BSL3.2 and describe several biophysical aspects of this RNA-RNA interaction. To do this, we fluorescently labeled 3′X55 with donor and acceptor fluorophores to monitor the inter-fluorophore distance of this RNA using single-molecule FRET (Fig. 1). At sub-nanomolar concentrations, individual fluorescently labeled RNA constructs diffuse through the sub-femtoliter confocal volume of our fluorescence microscopy system (Fig. 2*A*), where their fluorophores can be excited and subsequently emit “bursts” of fluorescence on the 1-ms timescale (Fig. 2*B*). Several thousand bursts were acquired over the course of 10 min. The donor and acceptor photons associated with each burst were then analyzed (Fig. 2*C*) to calculate transfer efficiency (*E*) values for FRET-labeled bursts, which were ultimately compiled into *E*-histograms to visualize the distribution of transfer efficiencies under a given set of experimental conditions.

### 3′X55 slowly interconverts between conformational states

**Figure 3:**
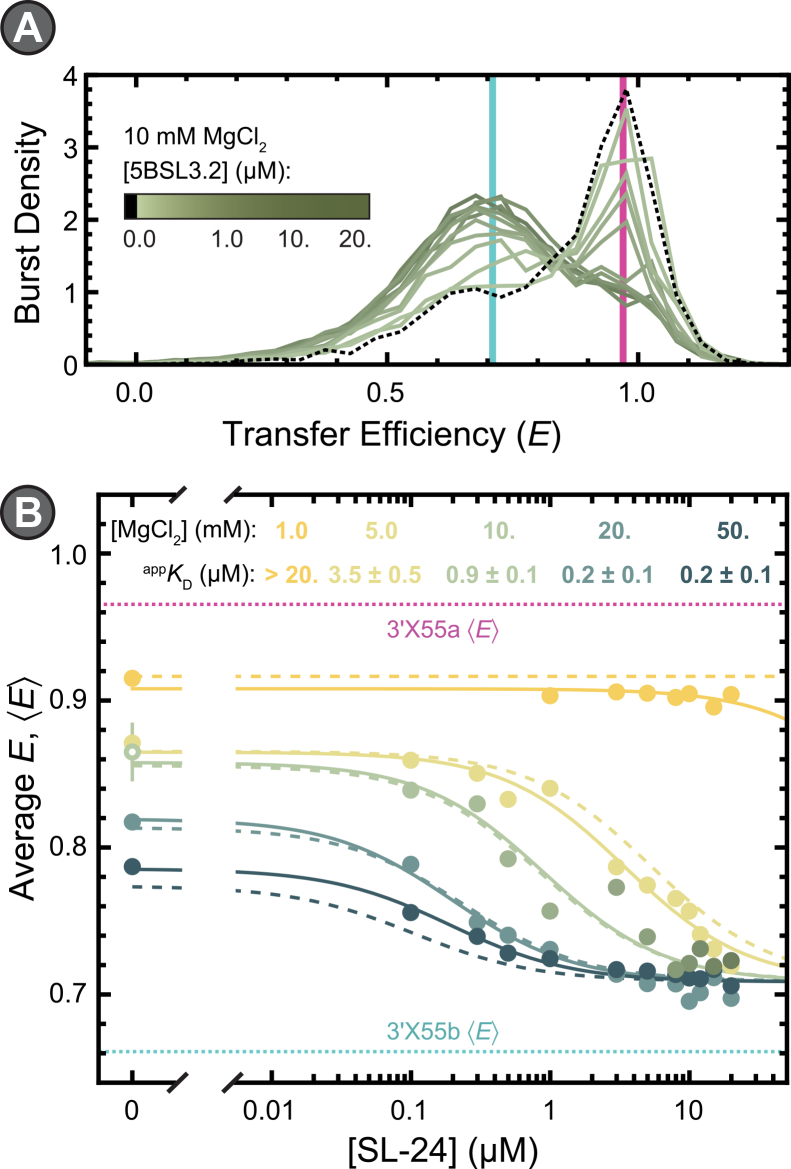
**The intramolecular conformational equilibrium of 3′X55 depends on 5BSL3.2.***A*, Overlaid *E*-histograms of 3′X55 in the presence of various concentrations of SL-24 under baseline experimental conditions. In the absence of SL-24 (*dashed black line*), 3′X55 predominantly adopts a high*-E* subpopulation associated with 3′X55a, while the less abundant intermediate*-E* bursts are associated with 3′X55b. The modes of the globally fit unbound subpopulations are depicted as vertical lines through the *E*-histograms (*cyan –* 3′X55b, *magenta* – 3′X55a). As SL-24 was added, the relative abundance of the high-*E* subpopulation decreased with a corresponding increase in the relative abundance of the intermediate*-E* subpopulation. As a result of these changes, the average transfer efficiency, ⟨*E*⟩, of 3′X55 decreased. *B*, SL-24-dependent ⟨*E*⟩ values were fit to an empirical binding model (*solid lines*), yielding the ^app^*K*_D_ under various concentrations of MgCl_2_ (see EXPERIMENTAL PROCEDURES). These measured ⟨*E*⟩ values were also compared to ⟨*E*⟩ derived from our four-state binding model (*dashed lines*), along with the ⟨*E*⟩ values associated with pure distributions of both 3′X55a and 3′X55b (*magenta* & *cyan*). The open circle in the 10 mM MgCl_2_ data set represents the average ⟨*E*⟩ value from eight independent replicate measurements in the absence of SL-24, and the vertical error bars represent the standard deviation of those measurements, which highlights typical measurement variability (±0.02).

Previous studies conducted in the absence of 5BSL3.2 (12) have shown that the first 55 nucleotides of 3′X (3′X55) slowly interconvert between two conformations: 3′X55a and 3′X55b (Fig. 1*A*). Consequently, samples containing 3′X55 take a long time to reach equilibrium after changing solution conditions (Fig. S2). Therefore, we ensured that the RNAs were fully equilibrated by conducting measurements at least 16 h after the samples were prepared. Our *E*-histograms obtained under baseline experimental conditions (see Experimental Procedures) depict two apparent subpopulations (Fig. 3*A* – *dashed, black*); one associated with the high-*E* (*∼* 1.0) conformation of 3′X55a and the other associated with the intermediate-*E* (∼ 0.7) conformation of 3′X55b. Importantly, the presence of both conformations under these conditions allows us to observe how each responds to the addition of 5BSL3.2.

### The intramolecular conformational equilibrium of 3′X55 depends on 5BSL3.2

To determine how the intramolecular conformational equilibrium of 3′X55 responds to the addition of 5BSL3.2, we prepared samples with increasing concentrations of SL-24, an unlabeled 24-nucleotide segment of 5BSL3.2 (Fig. 1*B* – *green*). Upon increasing the concentration of SL-24, we observed a systematic decrease in the relative abundance of the high-*E* subpopulation and a corresponding increase in the relative abundance of the intermediate*-E* subpopulation (Fig. 3*A*), suggesting that the dominant bound conformation closely resembles 3′X55b. To quantify the apparent binding affinity (^app^*K*_D_) of this interaction, we calculated the average transfer efficiency, ⟨*E*⟩, of 3′X55 as a function of SL-24 concentration and fit this data to an empirical binding model (Equation 3), resulting in a near-micromolar value for the ^app^*K*_D_ (Fig. 3*B*).

Next, we acquired similar datasets at four other MgCl_2_ concentrations to explore the magnesium-ion dependence of this RNA-RNA interaction (Fig. S3). Conveniently, the magnesium dependence of our measurements in the absence of SL-24 (Fig. S4) yields a half-maximal MgCl_2_ concentration that is in good agreement with a previously reported value for the intramolecular conformational equilibrium of 3′X55 (12). Conversely, our measurements in the presence of SL-24 yielded a notable decrease in the ⟨*E*⟩ values of 3′X55 (Fig. 3*B*), except in those measurements carried out at 1 mM MgCl_2_. Together, these results demonstrate that the intramolecular conformational equilibrium of 3′X55 synergistically depends on the concentration of both SL-24 and MgCl_2_. Furthermore, at MgCl_2_ concentrations ≥ 5 mM, 3′X55 can clearly be saturated with SL-24, as evidenced by the asymptote at ⟨*E*⟩ ≈ 0.7 (Fig. 3*B*). Curiously, under these saturating conditions, the ⟨*E*⟩ is notably larger than that associated with a pure population of 3′X55b because the high-*E* subpopulation remains present (Figs. 3 and S3). These observations are incompatible with a single bound state and suggest that this RNA-RNA interaction is likely governed by a binding mechanism that samples multiple bound states.

### A four-state binding model accurately describes the interactions between 3′X55 and 5BSL3.2

**Figure 4:**
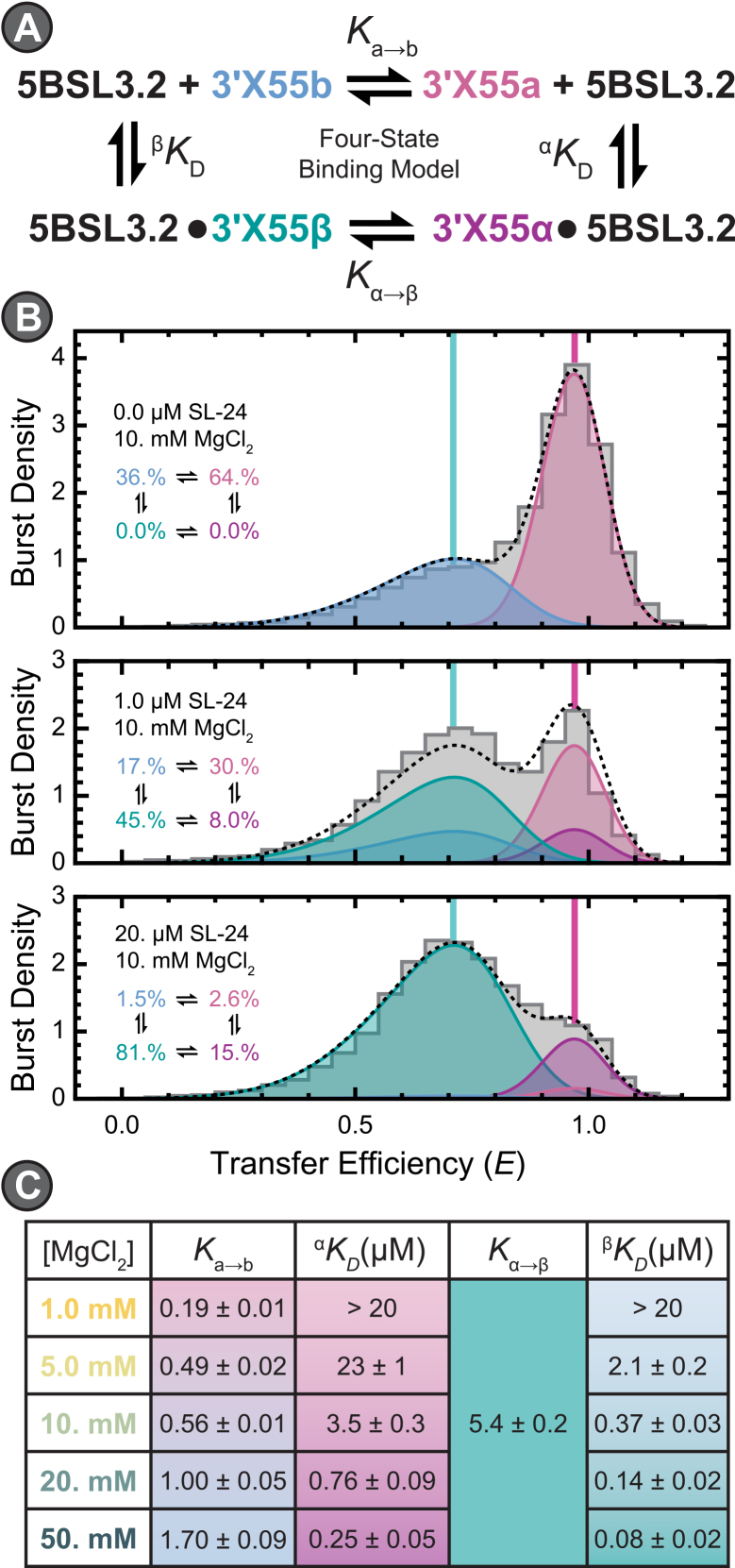
**Four-state binding model analysis.***A*, four-state binding model details the free interconversion of 3′X55 and the binding of 5BSL3.2 to 3′X55 within a network of four coupled equilibria (Fig. S1). *B*, representative *E*-histograms and the fitted distributions of the associated subpopulations resulting from our four-state binding model analysis. In the absence of SL-24 (*top*), the relative abundances of the free conformations dominate. As the concentration of SL-24 approaches saturation (*bottom*), we see that the relative abundances of the bound conformations dominate. *C*, table of equilibrium constants determined from the four-state binding model.

To more clearly resolve the mechanisms governing the interaction between 3′X55 and SL-24, we propose a system of four coupled equilibria (Figs. 4*A* and S1) that models interconversion among two free states (3′X55a and 3′X55b) and two bound states (3′X55α and 3′X55β). The equilibrium constants associated with this model were quantified by fitting each *E*-histogram (Fig. 4*B*) to a sum of four skewed Gaussian distributions, Ψ(E). These individual distributions, $\Psi_{i}\left(E\right)$ and their associated weights, $\delta_{i}$, describe the location, shape, and relative abundance of each subpopulation present within a given *E*-histogram (Fig. 4*B* − *solid lines*). We observed that the location and shape of the high- and intermediate-*E* subpopulations were largely invariant to changes in the concentration of SL-24 and MgCl_2_ (Fig. S3). Based on this observation, we chose to simplify our four-state binding model such that the 3′X55a and 3′X55α subpopulations are described by one set of shared location and shape parameters, and the 3′X55b and 3′X55β subpopulations are described by another set of shared location and shape parameters. The simplified model (Fig. 4*A*) was used to globally fit experimental *E*-histograms (Figs. 4*B* and S5*A*) to quantify the location and shape of each subpopulation (Fig. S5*B*), along with three equilibrium constants (Figs. 4*C* and S5*C*) that collectively describe the relative abundance of each subpopulation. This quantitative analysis reveals several notable insights that are highlighted below.

First, we observed that the equilibrium constant of bound 3′X55 (*K*_α*→*β_) was largely independent of the MgCl_2_ concentration, which motivated us to treat this as a single shared parameter. Second, *K*_α*→*β_ was consistently larger than the equilibrium constant of free 3′X55 (*K*_*a→b*_). Consequently, the binding affinity for SL-24 is conformation-dependent, with the equilibrium dissociation constant of 3′X55β (^β^*K*_*D*_) being consistently smaller than that of 3′X55α (^α^*K*_*D*_). Third, this analysis indicates that values of ^α^*K*_*D*_ and ^β^*K*_*D*_ both decrease as the concentration of MgCl_2_ increases, highlighting the importance of Mg^2+^ in the binding equilibria. Finally, to better assess our four-state binding model, we used the fitted parameters from the four-state binding model to derive the ⟨*E*⟩ as a function of SL-24 concentration (Equation 8) across all concentrations of MgCl_2_ (Fig. 3*B* – *dashed lines*). These derivations were in good agreement with both our experimental data and the fits from the empirical binding model (Fig. 3*B* – *solid lines*).

### 5BSL3.2 binding is largely insensitive to the structural context of the *k′-*sequence

**Figure 5:**
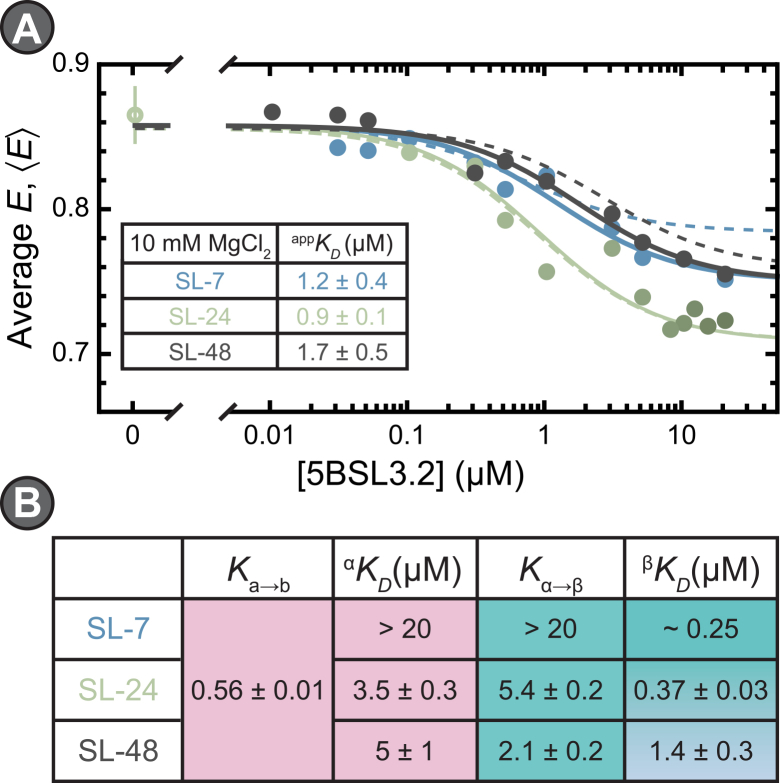
**3′X55 binds to structural derivatives of 5BSL3.2.***A*, Collated average transfer efficiency, ⟨*E*⟩, values from measurements of 3′X55 in the presence of the three 5BSL3.2 constructs were fit to empirical binding models (*solid lines*). These models were constrained to an initial ⟨*E*⟩ defined by averaging measurements in the absence of any 5BSL3.2 construct (*open circle*). Additionally, these measured ⟨*E*⟩ values were compared to the derived ⟨*E*⟩ values from our four-state binding model (*dashed lines*). *B*, table of equilibrium constants determined from our four-state binding model. Notably, all the 5BSL3.2 constructs have higher binding affinities for 3′X55b than 3′X55a.

The above experiments utilized SL-24 (Fig. 1*B* – *green*) to biochemically characterize the binding mechanism governing the interaction between 3′X55 and 5BSL3.2. To explore the structural context of this riboregulatory interaction, we generated two additional 5BSL3.2 constructs (Fig. 1*B*, Table S1): SL-48, which is a 48-nucleotide variant of 5BSL3.2; and SL-7, which is the 7-nucleotide *k′-*sequence of 5BSL3.2. Using these new constructs, we performed analogous measurements under baseline experimental conditions. Our data show that increasing concentrations of any of the three constructs increases the relative abundance of an intermediate-*E* subpopulation (Fig. S6). However, we note that the transfer efficiency of 3′X55β bound to SL-7 is notably higher than the transfer efficiency we observe when 3′X55β is bound to either of the other 5BSL3.2 constructs. Nevertheless, our SL-7 results indicate that the *k′-*sequence is largely sufficient for a near-micromolar ^app^*K*_D_ (Fig. 5*A*). Lastly, our analysis using the four-state binding model indicates that ^β^*K*_*D*_ is less than ^α^*K*_*D*_ for all three constructs (Fig. 5*B*), which further reinforces the notion that 3′X55b binds *k′-*sequence with a higher affinity than 3′X55a.

### 3′X55 structural mutant alters 5BSL3.2 binding mechanism

**Figure 6:**
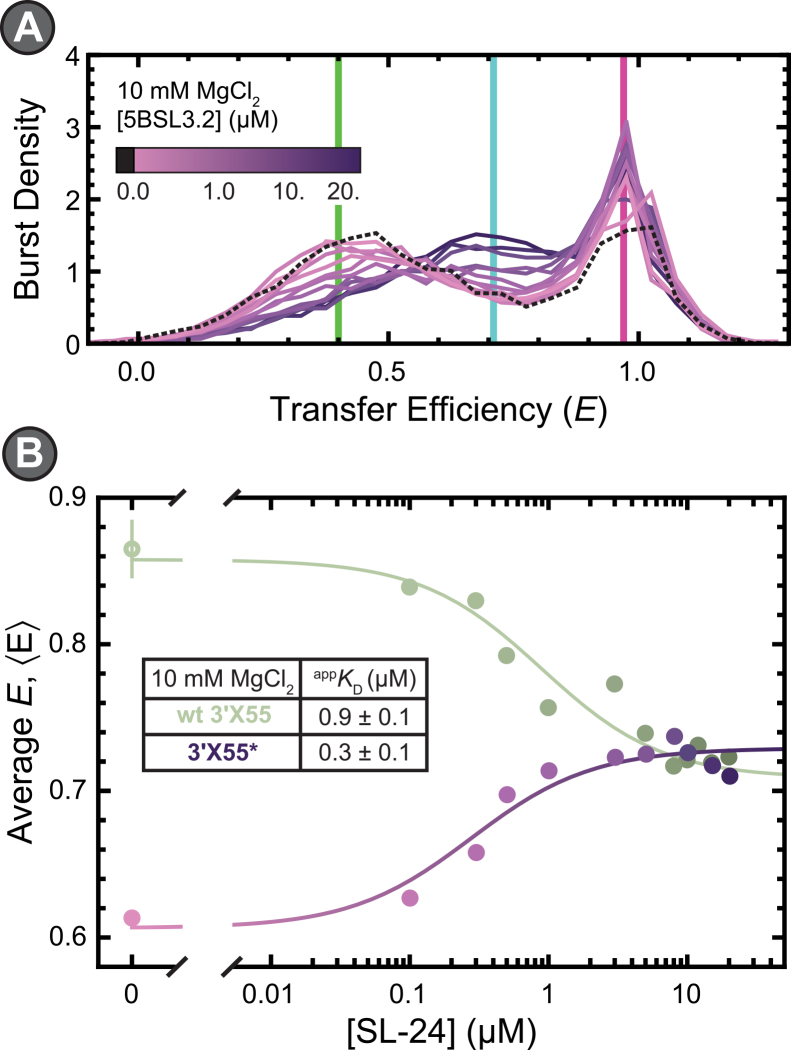
**3′X55∗ can bind 5BSL3.2.***A*, overlaid *E*-histograms of 3′X55∗ acquired at 10 mM MgCl_2_. As SL-24 is titrated into solution, 3′X55∗ adopts a wildtype-like distribution of transfer efficiencies, resulting in more similar average transfer efficiency, ⟨*E*⟩, values. *B*, all 3′X55∗ ⟨*E*⟩ values (*purple*) were fit to our empirical binding model (Equation 2, *solid line*), yielding ^app^*K*_D_ values. Wildtype 3′X55 ⟨*E*⟩ values (*green*) under the same conditions are shown for comparison.

Next, we set out to investigate the impact of a previously reported nucleotide substitution (A31C) (27), which happens to be located at one of the most highly conserved positions in 3′X. Like the wild-type sequence, this variant (3′X55∗) has two predicted secondary structures that are nearly isoenergetic. One of them is identical to 3′X55a; however, the other only retains the 6 base-pairing interactions associated with the first stem of 3′X55b (Fig. S7). This novel secondary structure is also predicted to yield ⟨*E*⟩ values that are lower than 3′X55b. Single-molecule measurements of fluorescently labeled 3′X55∗ support these predictions, yielding a high-*E* (∼ 1.0) subpopulation like that of 3′X55a, and a novel low-*E* (∼ 0.4) subpopulation in the absence of SL-24 (Fig. 6*A* – *dashed grey line*). Under baseline conditions, the addition of SL-24 introduced several changes to the *E*-histograms, including a pronounced decrease in the relative abundance of the novel low*-E* subpopulation, and an appearance of an intermediate-*E* (∼ 0.7) subpopulation like that of 3′X55β (Fig. 6*A*). Again, the ⟨*E*⟩ values of the 3′X55∗ measurements were fit to the empirical binding model to approximate the apparent binding affinity of this interaction (Fig. 6*B*). Despite the pronounced structural differences between the two RNAs, both have similar, low micromolar ^app^*K*_D_ values for SL-24 and asymptote at similar ⟨*E*⟩ values when bound. Here, however, we refrain from using any higher-order analyses as the novel low*-E* subpopulation associated with the incredibly unlikely mutation (A31C) in 3′X55∗ necessitates additional states beyond those present in our four-state binding model.

## Discussion

### 3′X55 has multiple binding modes

Our single-molecule measurements have enabled us to uncover how interactions with 5BSL3.2 alter the intramolecular conformational equilibrium between 3′X55a and 3′X55b. Previous NMR studies of 3′X independently observed interactions with 5BSL3.2 (11, 14, 15). However, these studies present differing conclusions regarding the structural context of this interaction (Fig. S1): some suggest that the *k-*sequence of 3′X55a locally melts to form a complex with 5BSL3.2 (14, 15), and others propose that this complex forms *via* direct interactions with the exposed *k-*sequence of 3′X55b (11). Here, we observed that adding 5BSL3.2 increases the relative abundance of the intermediate-*E* subpopulation, which suggests that the preferred bound conformation closely resembles 3′X55b. However, the high-*E* subpopulation remains present under saturating conditions (Fig. 4 and S3). This result is incompatible with any two-state model, so we propose that 3′X55 can interact with 5BSL3.2 in two distinct ways (Fig. S1). Under this four-state binding model, which supports interconversion between two free forms (3′X55a and 3′X55b) and two bound forms (3′X55α and 3′X55β) of 3′X55, both bound forms are present under saturating concentrations of 5BSL3.2. After analyzing our *E-*histograms with this four-state binding model, the resulting equilibrium constants indeed suggest that 3′X55a can bind 5BSL3.2, albeit with a much lower affinity than 3′X55b (Fig. 4, *B* and *C*). The decreased affinity for 3′X55a is likely because the paired *k*-sequence must first melt before it can associate with 5BSL3.2 (Fig. S1). Conversely, 3′X55b already has an unpaired *k*-sequence and can directly bind 5BSL3.2. Previous results from a surface plasmon resonance (SPR) study (28) show that 5BSL3.2 binds to a short, *k*-sequence containing oligonucleotide resembling 3′X55b. After rigorously inspecting these SPR results, we estimate that the affinity of this *k*-sequence containing oligonucleotide for 5BSL3.2 is a few micromolar, which is quite consistent with the low micromolar ^β^*K*_*D*_ we obtained under similar experimental conditions (*i.e.*, 3-5 mM MgCl_2_). Furthermore, the time scale of binding in these SPR studies was a few hundred seconds, which suggests that the processes of association/dissociation in our four-state binding model occur on a much faster time scale than the slow conformational interconversion between 3′X55a and 3′X55b (12) and between 3′X55α and 3′X55β (Fig. S2). Nevertheless, future studies using approaches that can independently report on intermolecular binding and intramolecular conformational exchange (*e.g.*, 3-color FRET) will be required to further refine this four-state binding model. However, it is important to remember that these are all bimolecular studies of a unimolecular intragenomic interaction, and the biologically relevant kinetics/energetics are likely dictated by the structure of the ∼ 300 nucleotides around/between these two structured elements located at the end of the HCV genome.

### Structural context of the *k*′-sequence subtly modulates binding

To assess how the *k-k*′ interaction depends on the structural context of the interacting oligonucleotides, we generated three 5BSL3.2 constructs of differing lengths: SL-7, SL-24, and SL-48 (Fig. 1*B*, Table S1). Our four-state binding model analyses of measurements involving these constructs demonstrate that all three bind to 3′X55. Furthermore, our analyses suggest that 3′X55b always binds 5BSL3.2 with a greater affinity than 3′X55a, further emphasizing the conformational preference for 3′X55β (Fig. 5*B*). However, the structural context of the *k′-s*equence does appear to moderately influence the relative stability of 3′X55α and 3′X55β. Specifically, we observed an increased conformation preference (*K*_α*→*β_) in the shorter 5BSL3.2 constructs. Such behavior could indicate that the paired nucleotides on either side of the *k′-s*equence in SL-24 and SL-48 give rise to conformational constraints that predominantly interfere with binding to the *k-s*equence of 3′X55b.

### 3′X55∗ binds 5BSL3.2 with novel mechanism

Additionally, we briefly explored the structural dependence of the *k-k*′ interaction *via* an RNA construct containing a single-nucleotide substitution in 3′X55. Because this non-coding RNA is so highly conserved, even seemingly benign mutations can disrupt its secondary structures. This mutant construct (3′X55∗) contains a non-viable A31C substitution, which has been used in previous studies to disrupt homodimerization (27). Although this contrived mutation does impede dimerization, here we show that it also alters several other structural aspects of this RNA that may have important functional consequences. Specifically, in the absence of 5BSL3.2, this mutation causes 3′X55 to adopt a novel secondary structure (Figs. 6*A* and S5). This observation highlights some of the nuances that must be considered when trying to study artificial variants of this highly conserved RNA. Nevertheless, we can confidently report that 3′X55∗ can still bind 5BSL3.2 (Fig. S6), which, in conjunction with our earlier results (Fig. 5), suggests that the *k-k*′ interaction is largely governed by sequence complementarity. In the presence of saturating concentrations of 5BSL3.2, the average transfer efficiency, ⟨*E*⟩, values of 3′X55 and 3′X55∗ are quite similar (Fig. 6*B*), indicating that binding of 5BSL3.2 induces a pronounced conformational change in 3′X55∗, resulting in a complex that closely resembles 3′X55β. This binding mechanism likely differs from that of 3′X55, which appears to bind in a manner more consistent with conformational selection (Fig. 3*A*). However, additional kinetic studies would be required to rigorously compare the binding mechanisms of these two RNAs.

### The intramolecular conformational equilibrium of 3′X may modulate aspects of both translation and replication

The highly conserved 3′X RNA of HCV is an essential riboregulatory element within the viral genome (6, 7). Its nucleotide sequence allows it to natively adopt two nearly isoenergetic monomeric conformations (12). Notably, these two conformations have differing affinities for 5BSL3.2, another regulatory element in the genome. We propose that when 5BSL3.2 binds to the high-affinity conformation, its ability to interact with the 5′UTR is restricted, leaving the IRES free to initiate cap-independent translation (16, 21) (Fig. 1*C* – *top*). Conversely, the low-affinity conformation of 3′X RNA is much less likely to associate with 5BSL3.2, leaving it free to function as an ideal template for (−) RNA synthesis *via* NS5B (12, 29) (Fig. 1*C* – *bottom*). This network of riboregulatory interactions involving three distinct regions of the HCV genome provides a mechanism to help separate the competing (and opposing) processes of translation and replication (18). Interestingly, the viral genome has a half-life of ∼ 2.7 h (30), which is comparable to the lifetimes of individual conformations of 3′X55 (12). This would suggest that during the early stages of viral infection, each copy of the genome is exclusively committed to either translation or replication. Furthermore, the conformational equilibria associated with this intragenomic riboregulatory network may contribute to the relative abundance of these functionally distinct forms of the viral genome.

## Conclusion

In this work, we characterized the interaction between two highly conserved elements within the RNA genome of the hepatitis C virus: 3′X and 5BSL3.2. Previous work demonstrates that 3′X favorably interacts with 5BSL3.2 and that 3′X slowly interconverts between two stable conformations. Using single-molecule FRET to characterize this RNA-RNA interaction, our results demonstrate that both conformations associated with the first 55-nucleotides of 3′X (3′X55) can bind to 5BSL3.2 and that binding modulates the intramolecular conformational equilibrium of 3′X55 to favor a particular bound conformation. These results appear to support an emerging hypothesis wherein 3′X and 5BSL3.2 are members of an intragenomic riboregulatory network that may influence viral translation and replication.

## Experimental procedures

### Conservation analysis

This work utilizes an HCV genotype 1b viral isolate (GenBank AJ238799.1) to define the reference sequences for 5BSL3.2 and 3′X. To determine the nucleotide conservation of these sequences, a BLAST + nucleotide database was generated containing all available HCV sequences deposited in the NCBI Nucleotide database under *H. hominis* (273,370 sequences as of August 31, 2024). Using the BLASTN-short algorithm, the database was queried with the reference sequences of 5BSL3.2 (nucleotides 9263–9311) and 3′X (nucleotides 9508–9605), resulting in 14,305 and 1482 pairwise alignments, respectively. The pairwise alignments were then imported into a Mathematica notebook, which filtered out a small number of pairwise alignments with low query coverage (qcov < 0.9), duplicate entries, and reverse transcripts. The filtered pairwise alignments were then exported as a FASTA file that was analyzed using MAFFT (version 7) (31) to create a single multiple sequence alignment (MSA) for each RNA. Both MSAs were then imported into a Mathematica notebook to determine the nucleotide conservation values for each position in the reference sequences (Figs. 1, *A*, *B* and 6*A*).

### Single-molecule FRET constructs

Several RNA oligos were used in this work (Table S1). The 3′X55 RNA oligo contains the first 55 nucleotides of 3′X and was synthesized, fluorescently labeled, and ligated as described previously (12). The 3′X55∗ RNA oligo is a variant of 3′X55 containing a single point mutation (A31C) and was prepared in the same manner as 3′X55. Both constructs were prepared from shorter RNA oligos that were fluorescently labeled at amino-modified sites (Table S1) with donor (Cy3B) and acceptor (CF660R) fluorescent probes containing NHS-ester reactive groups. Following reverse-phase HPLC purification (>90%), these donor and acceptor-labeled RNA oligos were covalently joined together *via* splinted ligation and again purified *via* reverse-phase HPLC. The resulting double-labeled RNA oligos were lyophilized, resuspended/refolded in storage buffer (10 mM HEPES, 5 mM NaOH, 0.1 mM EDTA), flash frozen in liquid N_2_, and stored at −80 °C until needed.

Additionally, three unlabeled 5BSL3.2 constructs were synthesized by Integrated DNA Technologies for this study (Table S1). SL-48 was the largest unlabeled construct and consisted of the entire 5BSL3.2 reference sequence (nucleotides 9263–9311) from GenBank AJ238799.1. Derivatives of this sequence were used to generate the other two constructs: SL-24 (nucleotides 9271–9294) and SL-7 (nucleotides 9281–9286). Once received, all three 5BSL3.2 constructs were suspended in storage buffer and verified for purity using UV absorbance spectroscopy. Individual aliquots were flash-frozen and stored at −80 °C until needed.

### Sample preparation

All single-molecule FRET samples contained 150 mM NaCl, 25 mM HEPES buffer, 12.5 mM NaOH, 50 μM Tween20, approximately 100 pM fluorescently labeled sample to achieve single-molecule conditions and varying concentrations of MgCl_2_, with baseline experimental conditions corresponding to 10 mM MgCl_2_ because it had an intermediate concentration of MgCl_2_ that was close to the half-maximal concentration of MgCl_2_ (Fig. S4). Additionally, samples contained varying concentrations of the 5BSL3.2 constructs. Once prepared, samples were left to equilibrate overnight at room temperature in the dark. Then, 90 μl of each equilibrated sample was transferred to a fresh well of an 18-well uncoated μ-slide (Ibidi).

### Single-molecule FRET measurements

All single-molecule FRET measurements were performed at 23 ± 0.5 °C on a modified inverted confocal fluorescence microscope (Fig. 2*A*, PicoQuant; MicroTime200) with two monochromatic pulsed excitation sources—515 nm (Omicron; Quixx 515-80PS) and 642 nm (Omicron; Quixx 642-140PS)—for the donor and acceptor fluorophores, respectively. The picosecond pulses (150 ps) from both diodes were coaxially aligned and adjusted to 90 μW while being modulated at 20 MHz in a Pulsed-Interleaved Excitation (32) configuration using a digital delay generator (Berkley Nucleonics Corporation; Model:577-4C). The overlapping beams were directed into the microscope by a multiband dichroic mirror (Chroma; ZT532/640rpc), before overfilling the back aperture of a 60 × water immersion objective (Olympus; UPlanSAPO). The diffraction-limited focus was positioned 50 μm into the solution above the top surface of the sample well. The fluorescence emitted from the excited donor and acceptor fluorophores is collected through the same objective and transmitted back through the multiband dichroic mirror. Donor and acceptor photons are focused through a 100 μm pinhole, split by polarization using a beam splitting cube (PicoQuant), and then spatially separated into distinct donor and acceptor photon streams using two long-pass dichroic mirrors (Chroma; T6351pxr). The four resulting photon streams are directed to individual avalanche photodiode detectors (Excelitas Technologies; SPCM-AQRH-14-TR), which pass photon detection events to a time-correlated single-photon counting module (PicoQuant; HydraHarp 400). All measurements were performed with a temporal resolution of 16 ps and were saved to .ptu files for analysis.

### Burst analysis

Measurement files (.ptu) were imported into Wolfram Mathematica 14.2 with a user-extendable software package, Fretica by Daniel Nettels, last compiled on November 9th, 2023 (https://github.com/SchulerLab/Fretica). First, all individual photons (Fig. 2*B*, *top*) are assigned to 1-s time bins, based on their arrival times at the detector (Fig. 2*B*, *bottom*). Any excessively bright 1-s time bins with a photon count that is more than three standard deviations above the mean photon count are excluded from the analysis as they are assumed to contain fluorescence from sources other than an individual fluorescently labeled RNA molecule freely diffusing in solution. All remaining photons are re-binned into 1-ms time bins, again based on their arrival times at the detector. Correction factors are applied to the collected photon counts to account for: (1) donor emission detected by acceptor detectors, (2) direct excitation of the acceptor fluorophore by the donor excitation source, (3) unequal detection efficiencies of photons, and (4) unequal excitation efficiencies of the fluorophores. After correction, bursts of fluorescence are identified as 1-ms time bins containing at least 30 photons (Fig. 2*B*). Next, average background counts, which are derived from all bins containing fewer than 30 photons, are subtracted from the identified bursts. Bins are then re-identified, as the background-corrected photon counts of each bin may have fallen below the 30-photon burst threshold. This identification process is repeated until the number of bursts identified remains constant. The photons from each burst are used to calculate two quantitative parameters (Fig. 2*C*). First, we calculate the fluorophore stoichiometry (*S*), where NTotal515 is the number of photons resulting from donor excitation and NTotal640 is the number of photons resulting from acceptor excitation (Equation 1). The value of *S* allows us to determine if a burst of fluorescence arose from a molecule where both the donor and acceptor were present and active, thus avoiding contaminating from the relatively small population (<3%) of donor- and acceptor-only molecules in the sample.

(Eq. 1) S=NTotal515(NTotal640+NTotal)515

Second, we calculate the transfer efficiency (*E*), where NAcceptor515 is the number of acceptor photons detected during donor excitation (Equation 2). The value of *E*, which experimentally measures the efficiency of Förster resonance energy transfer (FRET) (33), strongly depends on the inter-fluorophore distance and is used as a proxy for the conformational status of our fluorescently labeled 3′X55.

(Eq. 2) E=NAcceptor515NTotal515=11+(rR0)6

A fluorophore stoichiometry threshold (0.25 > S > 0.75) was used to maximize the number of FRET-active molecules in our analysis and to minimize contamination from donor- or acceptor-only molecules (Fig. 2*C*, *gold*). A threshold for the total number of photons (NTotal515+NTotal640>40) was chosen to ensure that the *E* values for every burst were determined using at least 13 photons after accounting for background and all relevant correction factors. In theory, the number of photons should only influence the width of each subpopulation and not its location or relative abundance. We felt that a value of 40 (13) made the two populations easy to resolve visually, while still maintaining enough bursts to obtain reasonably smooth-looking histograms containing > 1000 individual bursts in 10 min of measurement time. Values > 40 (13) yielded similar fitted parameters with more jagged-looking histograms comprised of fewer total bursts. Values < 40 (13) again yielded similar fitted parameters, but the resolution between the two populations was less obvious by visual inspection. Finally, these high-quality bursts were then compiled into *E*-histograms that are normalized by the total number of bursts and the bin width to approximate the empirical probability density function (PDF) of the distribution.

### Empirical binding model

To quantify the apparent binding affinity (KappD) of the various 5BSL3.2 constructs, population-averaged transfer efficiency, ⟨*E*⟩, values from individual measurements were fit to an empirical binding model (Equation 3).

(Eq. 3) ⟨E([5BSL3.2])⟩=(⟨E⟩final−⟨E⟩initial)[5BSL3.2][5BSL3.2]+KappD+⟨E⟩initial

Here, ${\left\langle E\right\rangle }_{initial}$ represents the ⟨*E*⟩ of 3′X55 in the absence of 5BSL3.2, ${\left\langle E\right\rangle }_{final}$ represents the ⟨*E*⟩ of 3′X55 under saturating 5BSL3.2 conditions, and KappD describes how the ⟨*E*⟩ changes as a function of 5BSL3.2 concentration *via* the traditional ligand-binding equation.

### Four-state binding model

We propose that the bimolecular interaction between 3′X55 and 5BSL3.2 can be more accurately modeled as a cyclic system of four subpopulations coupled by four equilibria (Fig. 4*A*), wherein 3′X55 interconverts between two free states (3′X55a and 3′X55b) and two bound states (3′X55α and 3′X55β). To determine the relative abundance of these four subpopulations (*i* = a, b, α, and β), each normalized *E*-histogram, Ψ(E), is fit to a weighted sum of four distributions (Equation 4), where the individual weights, $\delta_{i}$, quantify the relative abundance of the individual distributions, $\Psi_{i}\left(E\right)$.

(Eq. 4) $$\Psi \left(E\right)=\int \left(\sum_{i}{\;\delta }_{i}\Psi_{i}\left(E\right)\right)dE=1$$

The individual distributions, $\Psi_{i}\left(E\right)$, are empirically described by the PDF of a skewed Gaussian distribution (Equation 5).

(Eq. 5) $$\Psi_{i}\left(E\right)=\frac{1}{\sigma \sqrt{2\pi }}\;\exp\left(-\frac{{\left(E-\varepsilon \right)}^{2}}{2\sigma^{2}}\right)\;erfc\left(-\frac{\zeta \left(E-\varepsilon \right)}{\sigma \surd 2}\right)$$

Here, each $\Psi_{i}\left(E\right)$ has a location parameter, ε, that determines where the distribution resides in transfer efficiency space, and shape parameters, σ and ζ, that determine the width and asymmetry, respectively. Conveniently, when ζ=0 Equation 5 simplifies to the PDF of a canonical Gaussian distribution. The individual weights, $\delta_{i}$, are directly proportional to the relative concentrations of that subpopulation, assuming no major biases in burst identification. The ratios of these relative concentrations define the four equilibrium constants in the model (Equation 6).

$$K_{1}=\frac{\left[3^{\prime }X55a\right]}{\left[3^{\prime }X55b\right]}=\frac{\delta_{a}}{\delta_{b}}={\left(K_{a\to b}\right)}^{-1}$$

K2=[3′X55α][3′X55a][5BSL3.2]=δαδa[5BSL3.2]=(KDα)−1

$$K_{3}=\frac{\left[3^{\prime }X55\beta \right]}{\left[3^{\prime }X55\alpha \right]}=\frac{\delta_{\beta }}{\delta_{\alpha }}=\left(K_{\alpha \to \beta }\right)$$

(Eq. 6) K4=[3′X55b][5BSL3.2][3′X55β]=δb[5BSL3.2]δβ=(KDβ)

Conveniently, under the constraints of detailed balance (*i.e.*, $1=K_{1}\times K_{2}\times K_{3}\times K_{4}$) we can represent one of the equilibrium constants (*e.g.*, *K*_4_) as the inverse product of the others, reducing the number of fitted parameters. Finally, the weights can be rewritten in terms of the underlying equilibrium constants of this system (Equation 7).

$$\delta_{b}=\frac{1}{1+\left(K_{1}\right)+\left(K_{1}K_{2}\right)+\left(K_{1}K_{2}K_{3}\right)}$$

$$\delta_{a}=\frac{K_{1}}{1+\left(K_{1}\right)+\left(K_{1}K_{2}\right)+\left(K_{1}K_{2}K_{3}\right)}$$

$$\delta_{\alpha }=\frac{K_{1}K_{2}}{1+\left(K_{1}\right)+\left(K_{1}K_{2}\right)+\left(K_{1}K_{2}K_{3}\right)}$$

(Eq. 7) $$\delta_{\beta }=\frac{K_{1}K_{2}K_{3}}{1+\left(K_{1}\right)+\left(K_{1}K_{2}\right)+\left(K_{1}K_{2}K_{3}\right)}$$

Substituting Equations 5 and 7 into Equation 4 results in a fitting function for each *E*-histogram with three fitted parameters for equilibrium constants and three fitted parameters for each $\Psi_{i}\left(E\right)$. Collectively, the three equilibrium constants constrain the relative abundance of each 3′X55 subpopulation as a function of 5BSL3.2 concentration. For example, in the absence of 5BSL3.2 (when 3′X55 is completely free) the relative abundance is dictated solely by $K_{1}$. In contrast, in the presence of saturating concentrations of 5BSL3.2 (when 3′X55 is completely bound), the relative abundance is dictated solely by $K_{3}$. Naturally, a single *E*-histogram would not be sufficient to estimate all 15 parameters with confidence. To overcome this limitation, the location and shape parameters (ε, σ, and ζ) associated with the four subpopulations are assumed to be the same for the free populations (3′X55a and 3′X55b) and for the bound populations (3′X55α and 3′X55β), and these parameters are shared across all measurements. Additionally, globally fitting all *E*-histograms (Fig. S5) allows us to more robustly determine equilibrium constants (Figs. 4 and 5) given that they are shared across all measurements conducted under the same experimental conditions (*e.g.*, MgCl_2_ concentration), regardless of the 5BSL3.2 concentration. Finally, we utilize Equation 4 to derive the average transfer efficiency, ⟨*E*⟩, from the fitted parameters (Equation 8), allowing us to compare our four-state binding model to our empirical binding model and our experimental data.

(Eq. 8) $$\int \left(\sum_{i}{\;\delta }_{i}\Psi_{i}\left(E\right)\right)EdE=\left\langle E\right\rangle$$

### Modeling of average transfer efficiency values

The nucleotide sequences of 3′X55 and 3′X55∗ in conjunction with bracket-dot maps of the associated secondary structures (Figs. S1 and S6) were fed to RNAComposer (34), which utilizes machine translation principles in conjunction with the RNA FRABASE database to generate three-dimensional structural predictions. The .pdb files of these predictions were then passed to the FRET Positioning and Screening software (35), which determines accessible volumes of our fluorophores at their specific labeling positions based on rigid body docking. The accessible volumes of both the donor and acceptor fluorophores are then used to calculate the mean inter-fluorophore distance, which is used to model the average transfer efficiency of these three-dimensional structural predictions (Fig. S1) *via* the Förster equation (Eq. 2), assuming an R_0_ of 5.9 nm.

## Data availability

Data supporting the findings of this manuscript are available from the corresponding authors upon reasonable request.

## Supporting information

This article contains supporting information (8, 9, 15).

## Conflict of interest

The authors declare that they have no conflicts of interest with the contents of this article.
