# Supplementary material for: Intragenomic riboregulatory interaction modulates the conformation of the hepatitis C virus 3′X RNA
Source: J Biol Chem. 2026 Jun 19;302(8):113264. doi: 10.1016/j.jbc.2026.113264 (PMC13400342; doi:10.1016/j.jbc.2026.113264)
Supplement: Supporting Table and Figures [file mmc1.docx]

Intragenomic riboregulatory interaction modulates the conformation of the hepatitis C virus 3′X RNA

Ethan P. Rogers^1^, Parker D. Sperstad^1^, Erik D. Holmstrom^1,2*^

^1^ - University of Kansas Department of Molecular Biosciences

^2^ - University of Kansas Department of Chemistry

^*^ - For correspondence: Erik D. Holmstrom, edh@ku.edu

### Table S1: 5BSL3.2 Nucleotide sequences:

| Construct (nucleotide range) | Sequence (5′‑3′) |
| --- | --- |
| 3′X55 (9508‑9562) | GGUGGCUCCAUC/iAmC6T/UAGCCCUAGUCACGGCUAGCUG UGAA/iUniAmM/AGGUCCGUGAGCCGCU |
| 3′X55* (9508‑9562) | GGUGGCUCCAUC/iAmC6T/UAGCCCUAGUCACGGCUCGCUG UGAA/iUniAmM/AGGUCCGUGAGCCGCU |
| SL‑7 (9281‑9286) | UCACAGC |
| SL‑24 (9271‑9294) | GAGACAUAUAUCACAGCCUGUCUC |
| SL‑48 (9263‑9311) | CAGCGGGGGAGACAUAUAUCACAGCCUGUCUCGUGCCCGACCCCGCUG |

#### /iAmC6T/ = donor fluorophore labeling site

#### /iUniAmM/ = acceptor fluorophore labeling site

####
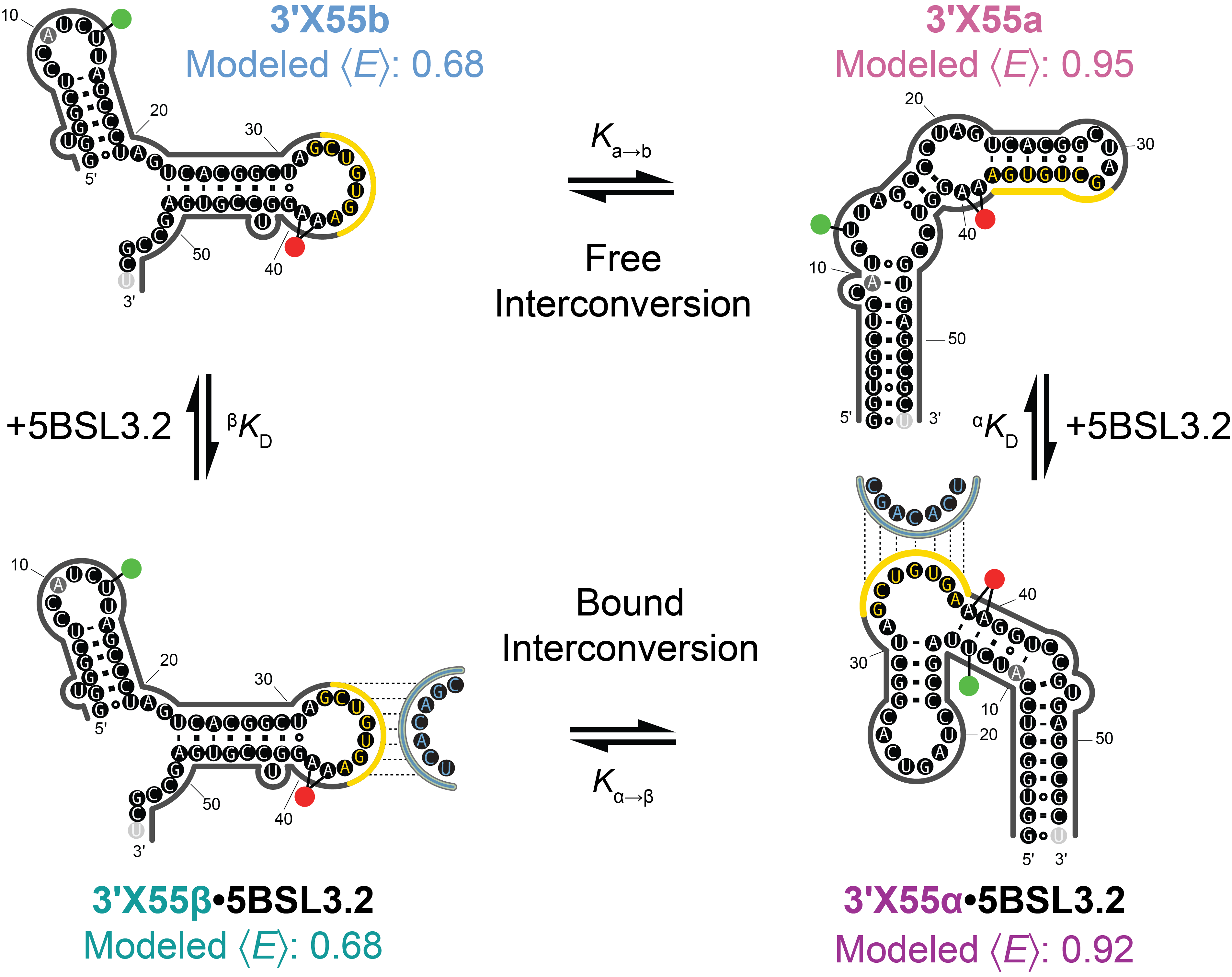


### Figure S1: Proposed 2° structures of 3′X55 & modeled transfer efficiencies

#### 3′X55 adopts two distinct monomeric conformations in the absence of 5BSL3.2, which can slowly interconvert. In the presence of 5BSL3.2, 3′X55 can pair its *k*-sequence (*yellow outline*) to the complementary *k′*-sequence of 5BSL3.2 (*blue outline*). The secondary structures of free 3′X55a and free 3′X55b were taken from previously published reports (Blight and Rice, *JVI*, 1997; Cantero-Camacho and Gallego, *NAR*, 2015). The proposed secondary structures of 3′X55α and 3′X55β were predicted via Mfold v2.3 at 20 °C by constraining *k*-sequence nucleotides to be unpaired so they can interact with *k*′. Notably, 3′X55α is consistent with the results of a previous NMR study of the 3′X55-5BSL3.2 complex (Castillo-Martinez et al., *NAR*, 2022).


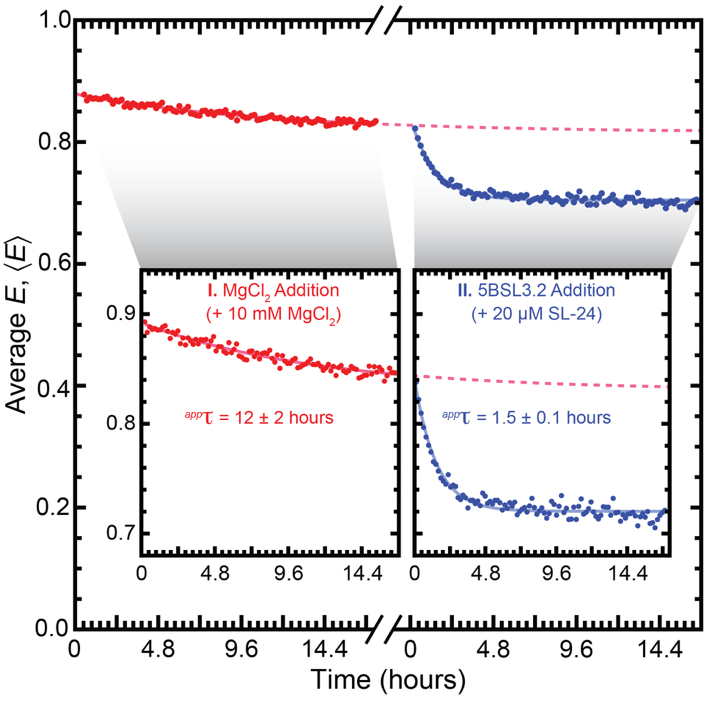


### Figure S2: Time dependence of conformational exchange

#### Sample preparation for kinetics measurements followed the same protocol as that used for equilibrated measurements (see EXPERIMENTAL PROCEDURES). In the first set of measurements (*red*), 3′X55 was allowed to equilibrate to baseline conditions (i.e., 150 mM NaCl, 25 mM HEPES buffer, 12.5 mM NaOH, 50 µM Tween20, and 10 mM MgCl_2_). After the addition of MgCl_2_, 50 µL of the sample was immediately added to a 15‑well angiogenesis µ‑slide (Ibidi), capped to prevent evaporation, and placed on the microscope using a low-vapor pressure immersion oil mimicking the refractive index of water. (Zeiss, ImmersolW). Data were collected in 102 consecutive 10‑minute measurements. Addition of MgCl_2_ stabilizes 3′X55b, decreasing the average transfer efficiency, 〈*E*〉, over time. In the second set of measurements (*blue*), 3′X55 pre-equilibrated to baseline conditions was allowed to equilibrate to the addition of SL‑24 (20 µM), which was also pre-equilibrated to baseline conditions. Addition of SL‑24 stabilizes 3′X55b, further decreasing the average transfer efficiency, 〈*E*〉, over time. Time-dependent 〈*E*〉 values from both kinetics measurements were fit to exponential decay functions of the following form to characterize the apparent time constant, ^app^*𝜏*, governing the conformational exchange of 3′X55 under the specified solution conditions:

#### $E(t)={(e^{\frac{-t}{{}^{app}\tau}}(\left\langle E \right\rangle}_{initial}-\left\langle E \right\rangle_{final}))+\left\langle E \right\rangle_{final}$.


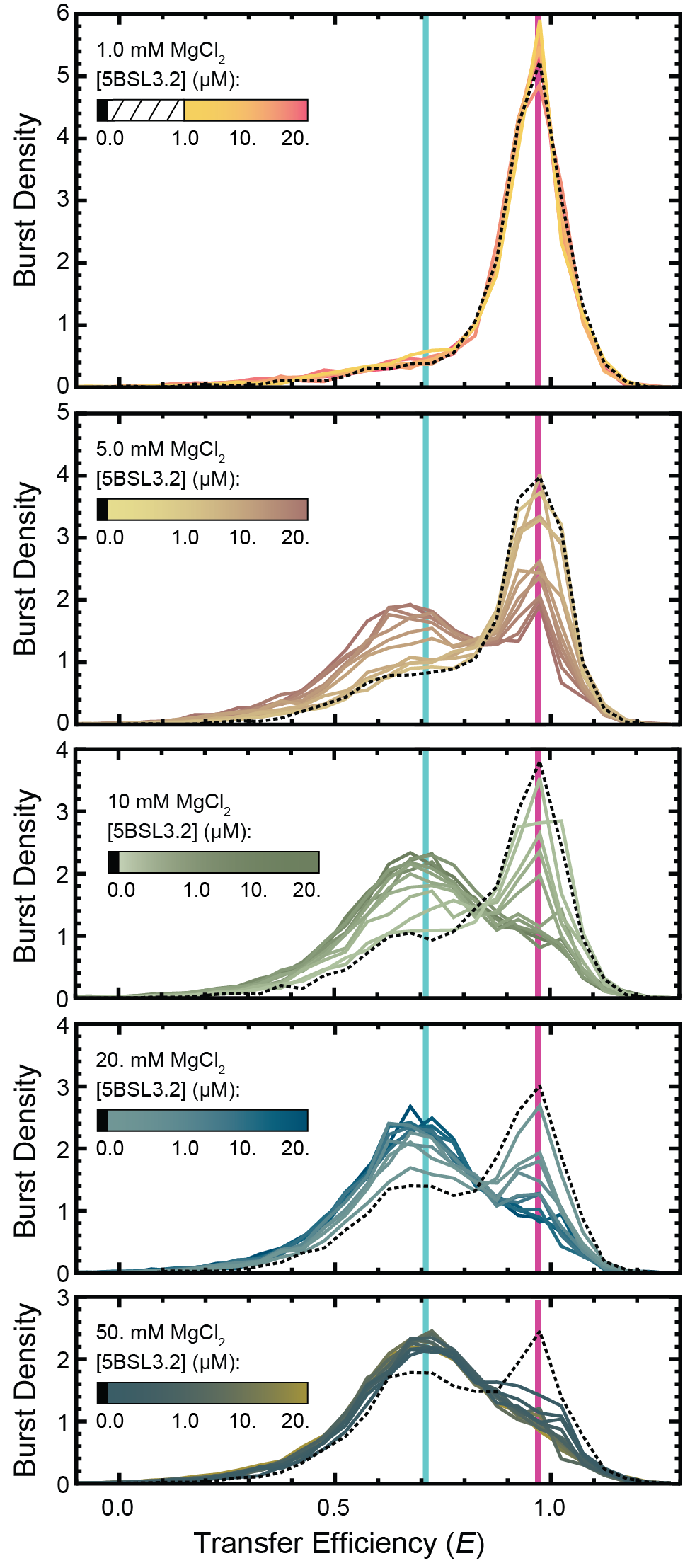


### Figure S3: SL‑24 binding measurements

#### Overlaid 3′X55 *E*-histograms acquired at 1, 5, 10, 20, and 50 mM MgCl_2_. In the absence of SL‑24 (*dashed,* *black*), 3′X55 predominantly adopts a high*‑*transfer efficiency (*E*) subpopulation associated with 3′X55a at all MgCl_2_ concentrations, although the relative abundance of this subpopulation decreases as the concentration of MgCl_2_ increases*.* As SL‑24 was added, the relative abundance of the high‑*E* subpopulation decreased with a corresponding increase in the relative abundance of the intermediate*‑E* subpopulation. This redistribution of burst density results in a decrease in the average transfer efficiency, 〈*E*〉. Even at 50 mM MgCl_2_ (*bottom*) with saturating SL‑24, the high‑*E* subpopulation remains. This observation is incompatible with a single bound state and suggests that this RNA‑RNA interaction is likely governed by a binding mechanism that samples multiple bound states. The modes of the unbound subpopulations are depicted as vertical lines through the *E*-histograms (*cyan –* 3′X55b, *magenta* – 3′X55a).


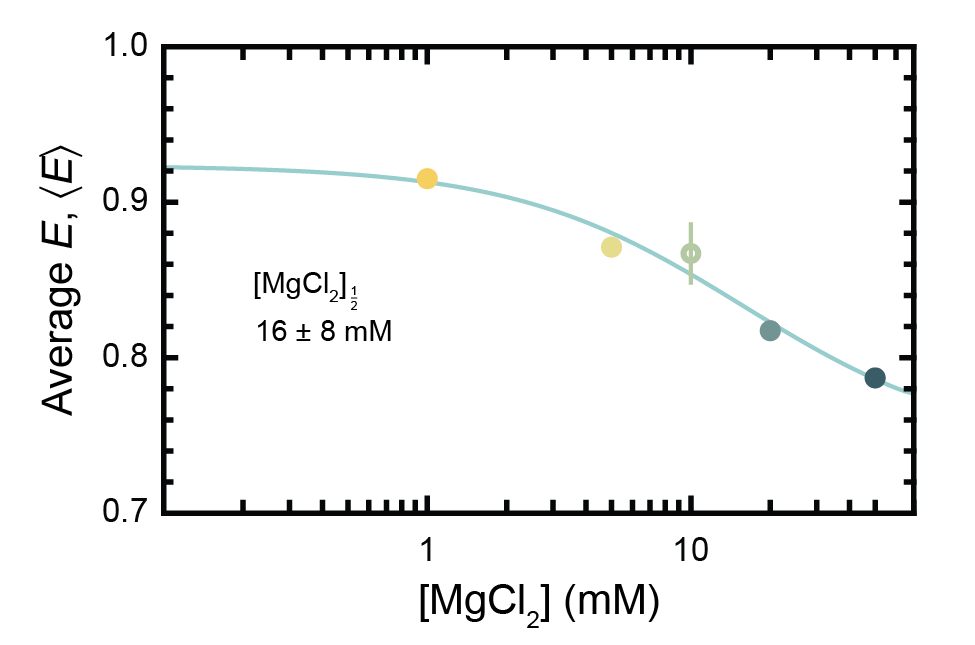


### Figure S4: MgCl_2_ dependence of unbound 3′X55

The average transfer efficiency, 〈*E*〉, of 3′X55 was determined from single‑molecule FRET measurements under various concentrations of MgCl_2_ in the absence of SL‑24. These data were fit to a modified form of our empirical binding model to determine the half-maximal concentration of MgCl_2_, $\left[ \mathrm{MgCl}_{2} \right]_{\frac{1}{2}}$.


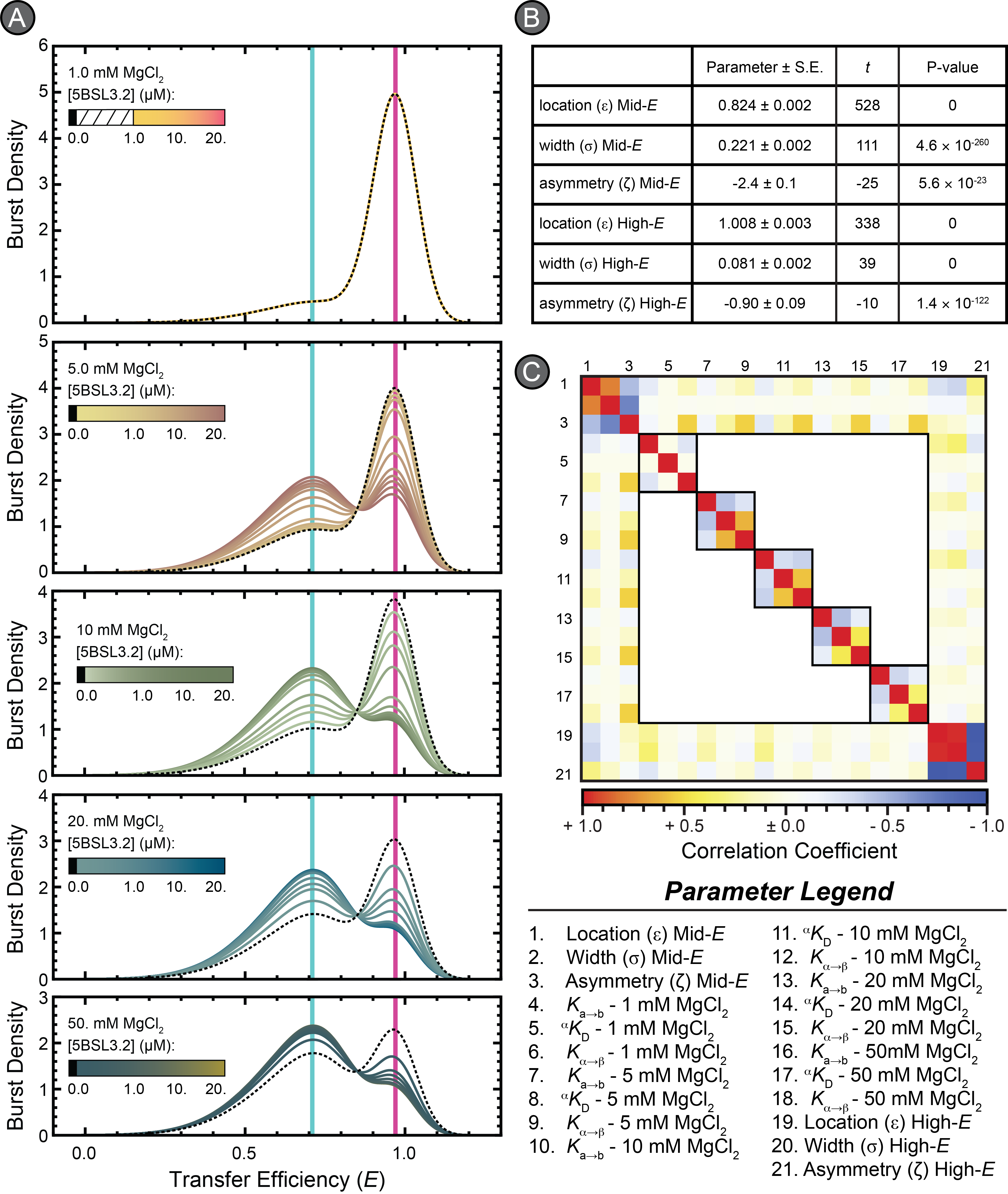


### Figure S5: Four-state model parameter analysis

**(A)** Overlaid fits from the four-state binding model analysis for SL‑24 at all MgCl_2_ concentrations (**Eq. 4**). The modes of the unbound subpopulations are depicted as vertical lines through the overlaid fits (*cyan -*3′X55b, *magenta* - 3′X55a). **(B)** Table of fitted values for peak location and shape parameters (**Eq. 5**). Equilibrium constant parameters are shown in **Figure 4C**. In all instances, the uncertainty represents the standard error from a non-linear model fit. **(C)** Correlation matrix of all fitted parameters.

### Figure S6: Measurements with structural derivatives of 5BSL3.2

#### Overlaid 3′X55 *E*-histograms acquired at 10 mM MgCl_2_ with various concentrations of the three different 5BSL3.2 constructs*.* As the 5BSL3.2 constructs were added, the relative abundance of the high‑*E* subpopulation decreased with a corresponding increase in the relative abundance of an intermediate*‑E* subpopulation associated with 3′X55β. The modes of the unbound subpopulations are depicted as vertical lines through the *E*-histograms (*cyan –* 3′X55b, *magenta* – 3′X55a).


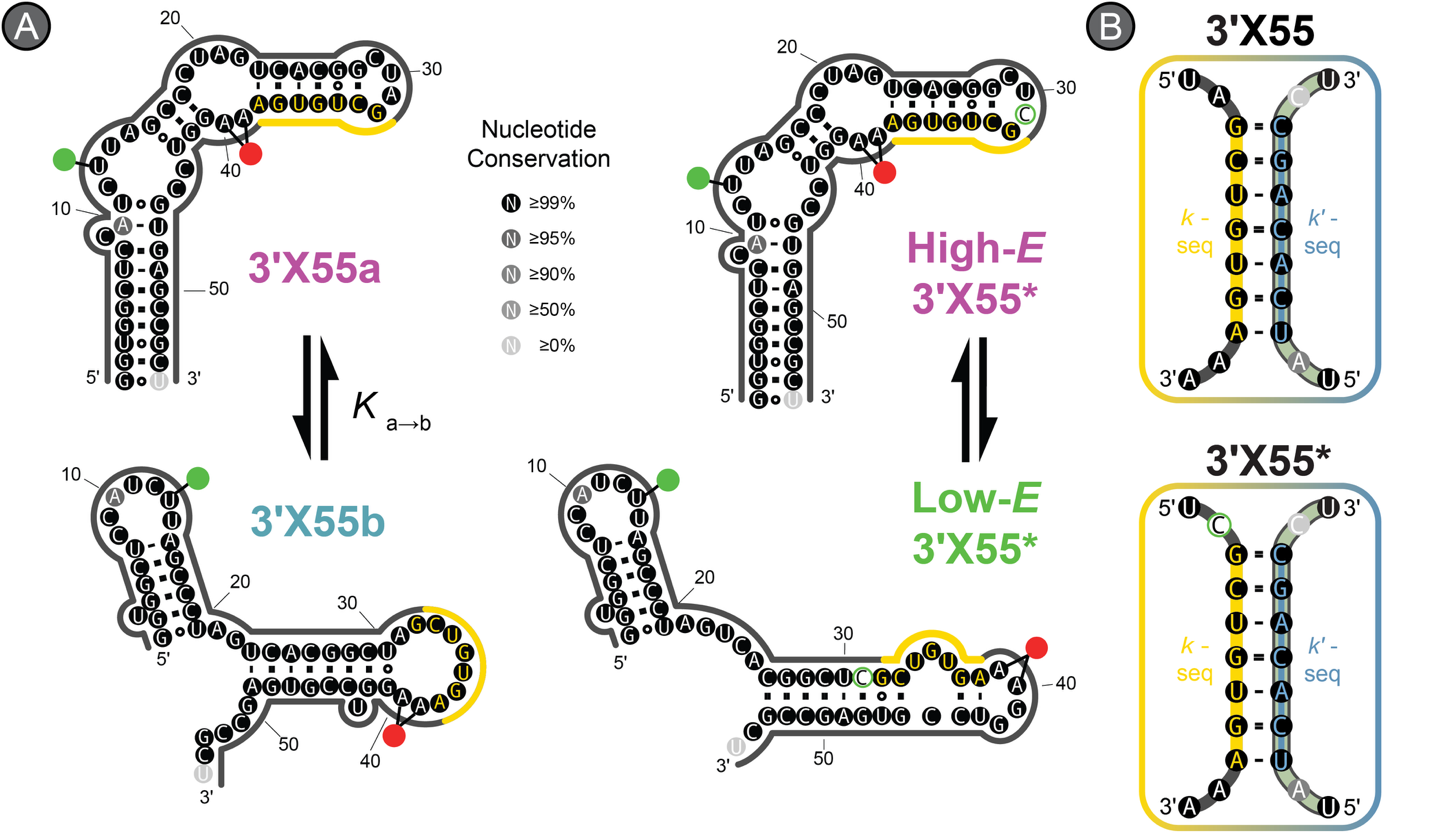


### Figure S7: Predicted structures of 3′X55*

#### **(A)** Structures of 3′X55* were generated by Mfold v2.3 at 20 °C. One of them is structurally identical to 3′X55a, while the other is a conformation that only retains 6 of the 14 base-pairing interactions associated with 3′X55b. This low transfer efficiency population has an extended stem in nucleotides 26-53, in contrast with the wildtype, whose stem progresses from nucleotides 22-49. **(B)** As this A31C mutation is outside of the *k*-sequence, the capacity to bind 5BSL3.2 remains, regardless of the novel conformation 3′X55* adopts.
